# Clinical and metabolic phenotypes of Oxford Biobank subjects with variations in human flavin-containing monooxygenase 5 (*FMO5*)

**DOI:** 10.1007/s11306-025-02308-1

**Published:** 2025-09-09

**Authors:** Jeremy R. Everett, Fredrik Karpe, Adrien Le Guennec, Matt Neville, Christina Redfield

**Affiliations:** 1 Medway Metabonomics Research Group, University of Greenwich, Chatham Maritime, Kent, ME4 4TB UK; 2 Oxford Centre for Diabetes, Endocrinology and Metabolism, University of Oxford, Oxford, OX3 7LE UK; 3 NIHR Oxford Biomedical Research Centre, OUH Foundation Trust, Oxford, OX3 9DU UK; 4 NMR Facility, King’s College London, Hodgkin Building, Guy’s Campus, London, SE1 1UL UK; 5 Department of Biochemistry, University of Oxford, South Parks Road, Oxford, OX1 3QU UK

**Keywords:** *FMO5*, Metabonomics, Metabolomics, NMR spectroscopy, Human clinical, Genetics, Heterozygous coding variations

## Abstract

**Introduction:**

Knockout of the *Fmo5* gene in mice led to a lean, slow-ageing phenotype characterised by the presence of 2,3-butanediol isomers in their urine and plasma. Oral treatment of wildtype mice with 2,3-butanediol led to a low cholesterol, low epididymal fat phenotype.

**Objectives:**

Determine if significant, heterozygous coding variations in human *FMO5* would give rise to similar clinical and metabolic phenotypes in humans, as in C57BL/6J mice with knockout of the *Fmo5* gene and in particular, increased excretion of 2,3-butanediol.

**Methods:**

Recruitment of 12 female, Oxford Biobank volunteers with heterozygous coding variations in *FMO5*, associated with changed clinical traits, and 12 age- and gender-matched controls. Analysis of the key NMR-based, urine and plasma, metabolic phenotypes of these volunteers to determine if there were any statistically significant differences.

**Results:**

Some clinical parameters of the female volunteers with heterozygous coding variations in *FMO5* were altered in a direction consistent with our hypothesis viz; lower insulin levels and lower waist circumference, but no consistent elevation of urinary 2,3-butanediol was found in the subjects with heterozygous coding variations in *FMO5*.

**Conclusion:**

Heterozygous coding variations in human *FMO5* appeared to have some impact on the clinical phenotype of the females in this study but the natural variation in the levels of 2,3-butanediol was higher than any inter-group differences between women with heterozygous coding variations in human *FMO5* and the women in the control group with wildtype *FMO5*.

**Supplementary Information:**

The online version contains supplementary material available at 10.1007/s11306-025-02308-1.

## Introduction

Metabolic profiling or metabonomics, also known as metabolomics, has emerged as an important method for understanding the molecular phenotypes of animals, plants and other complex biological systems (Lindon et al., 2000; Everett et al., 2019). The method is powerful, as through the observation of animal and plant metabolite profiles, it integrates genomic and environmental influences on the clinical phenotype, including the microbiome, in a systems biology approach, that can lead to prognostic as well as diagnostic information (Clayton et al., 2006, 2009). The methodology employs either hyphenated mass spectrometry (GC-MS, HPLC-MS, UPLC-MS; McCullagh & Probert, 2024; Zhang et al., 2020) or nuclear magnetic resonance spectroscopy (NMR; Emwas et al., 2019; McCullagh & Probert, 2024; Wishart et al., 2022) to detect, identify and quantify metabolites in a variety of biological matrices, often with advanced statistical methods to analyse the complex data (Misra, 2021; Worley & Powers, 2013).

The age-related, lean phenotype of the flavin-containing monooxygenase 5 knockout (*Fmo5*^*−/−*^) mouse is characterised by slow metabolic ageing, low body mass, low plasma cholesterol and less fat in white adipose tissue, in spite of greater food intake, relative to wildtype C57BL/6J (WT) mice (Gonzalez Malagon et al., 2015). Metabonomics showed that *Fmo5*^*−/−*^ mouse urine is characterised by the presence of two isomers of 2,3-butanediol (23BD); meso and enantiomeric (almost certainly the 2R, 3R but not known unambiguously). These two metabolites are produced by the altered gut microbiome of the mouse, as a result of the loss of FMO5, now known to be involved in bacterial sensing in the mouse and human gut (Scott et al., 2017). Reduction of 23BD levels through antibiotic treatment of *Fmo5*^*−/−*^ mice leads to an elevation in cholesterol back to WT mouse levels (Veeravalli et al., 2022). Treatment of WT mice with 23BD leads to significant lowering of plasma cholesterol and epididymal fat deposition but not body mass (Veeravalli et al., 2022).

In this study, the urine and plasma from humans with significant heterozygous coding variations in their *FMO5* gene was compared with urine and plasma from humans without *FMO5* coding variations using nuclear magnetic resonance spectroscopy (NMR) (Dona et al., 2016). The aims of the study were as follows:

1. Determine if humans with heterozygous coding variations in *FMO5* had a different clinical phenotype from those with wildtype *FMO5*
2. Determine if humans with heterozygous coding variations in *FMO5* excreted higher levels of 23BD than their counterparts with wildtype FMO5, in translational analogy to the *Fmo5*^*−/−*^ mice
3. Determine if other metabolic differences observed between *Fmo5*^*−/−*^ and WT mice were present in humans with heterozygous *FMO5* coding variations

Successful demonstration of translation of metabolic and clinical phenotypes from *Fmo5*^*−/−*^ mice to humans with coding variations in *FMO5* would be significant as it would give more confidence that dosing humans with 23BD could lower cholesterol and body fat, as observed when dosed in WT mice.

## Materials and methods

### Ethical approval

Full ethical approval for the study was held by the Oxford Biobank under Professor Fredrik Karpe (IRAS ID 329964; REC Name South Central—Oxford C Research Ethics Committee; REC Reference 23/SC/0411).

In addition, local approval was obtained from the University of Greenwich Ethics Committee for the temporary storage and analysis of the samples: UREC Application 21.4.74; approved 14.08.22.

### Sample collection and subject genomic and phenotypic data

Study volunteers were recruited from the Oxford Biobank (Karpe, et al., 2018, PMID: 29040543, https://www.oxfordbiobank.org.uk), a population-based cohort of healthy 25–55-year-old subjects from Oxfordshire who have undergone detailed metabolic phenotyping, UK Biobank Axiom® Array genotyping and provided consent to be recalled by phenotype or genotype for additional clinical studies. An initial linear regression analysis of genetic data for 7086 individuals in the Oxford Biobank within the FMO5 genomic region was undertaken to identify any genetic associations with phenotype or biochemical parameters held. Of the 18 SNPs within this region screened two were identified with female specific associations (Table 1), therefore recruitment was restricted to females only. Female participants heterozygous for the rs58351438 and rs143837136 SNPs and wildtype individuals age matched within 10 years were invited in to give a blood and urine sample. Heparin plasma and urine samples were then blinded before releasing to the researchers.

**Table 1:** Clinical Characteristics of the Female Subjects with the rs58351438 and rs143837136 FMO5 variants

A						
rs58351438						
Measure	n*	Het Count	Beta	SE	p**	% change
Insulin	4003	71	−0.04	0.01	0.002	12.40%
Waist	4001	71	−0.02	0.01	0.006	1.60%
Total android fat	2621	51	−0.02	0.01	0.023	10.20%
Visceral fat	2610	52	−0.04	0.02	0.014	48.60%
Subcutaneous android fat	2610	51	−0.01	0.01	0.266	8.40%

B						
rs143837136						
Measure	n*	Het Count	Beta	SE	p**	% change
Insulin	4003	12	−0.046	0.014	0.001	29.12%
Waist	4001	12	0.004	0.008	0.645	0.95%
Total android fat	2621	7	0.014	0.010	0.163	17.76%
Visceral fat	2610	7	−0.002	0.015	0.911	35.75%
Subcutaneous android fat	2610	7	0.013	0.011	0.257	11.33%

*The total count, n, is for females only, with Het Count being the number of case subjects with the SNP, Beta is the standardised measure of the effect size, as calculated in PLINK (https://zzz.bwh.harvard.edu/plink/) with the’Standard-beta’ modifier, and SE is the standard error

**p values are based on a linear regression model and adjusted for age and BMI

### Sample processing

Human urine and plasma samples were transported frozen in solid CO_2_ to the University of Greenwich and stored at −80 °C until prepared for analysis. The samples were supplied blinded and in triplicate.

600 ul of each urine sample was mixed with 100 ul pD 7.4 phosphate buffer made up from K_2_HPO_4_ (1.04 g, Fisher) and Na_2_H_2_PO_4_ (0.93 g, Fisher) in 100% D_2_O (10 ml, Sigma) with 3-trimethylsilyl-2,2,3,3-propionate-d_4_ reference (0.86 mg, Sigma). The samples were vortexed for 15 s to ensure good mixing and then centrifuged at 4 °C and 13,000 g for 4 min. 600 ul of each sample was then aspirated into a 5 mm outside diameter SampleJet NMR tube (Bruker) and placed in a SampleJet rack (Bruker). After issues with tuning, obtaining good lineshape and resolution on the Oxford 950 NMR spectrometer, ca 200 ul of each of the urine samples was transferred into 3 mm SampleJet tubes (Bruker). No tuning, lineshape or resolution issues were encountered and the NMR analyses were then conducted in these tubes. 600 ul of each heparin plasma sample was mixed with 100 ul of 100% D_2_O (10 ml, Sigma) and then processed as for the urine samples above.

After preparation, samples were kept at 4 °C until analysis both during storage and transport to the NMR facility. No sample degradation was observed. Samples were kept at 4 to 7 °C in the Sample Jet Sample Changer (Bruker) until analysis. Just prior to insertion in the magnet, samples were pre-heated to 300 K and then analysed at 300.0 K (urine) and 310 K (plasma).

### NMR spectroscopy

NMR Data was acquired at a proton frequency of 700 MHz (University of Southampton and Kings College London (KCL)) and at 950 MHz (University of Oxford, urine only). One-dimensional (1D) ^1^H NMR spectra were acquired with a noesy water presaturation sequence with gradient pulses; Bruker experiment noesygppr1d. In addition, Carr-Purcell-Meiboom-Gill spin echo spectra were acquired for the plasma samples using the Bruker pulse sequence cpmgpr1D. Two-dimensional (2D) correlation spectroscopy (COSY), total correlation spectroscopy (TOCSY), heteronuclear single quantum correlation spectroscopy (HSQC) and heteronuclear multiple bond correlation spectroscopy (HMBC) experiments were performed with the standard Bruker experiments: dipsi2gpphpr, hsqcetgpsisp2.2 and hmbcgplpndprqf respectively, all with water signal suppression and gradient pulses.

NMR data were analysed using MNova version 12.0.1–20,560 (Mestrelab, 2018). One-dimensional ^1^H NMR data were zero-filled from 64 to 128 K data points and sensitivity-enhanced by exponential multiplication of the free induction decay with line-broadening, LB = 0.3 Hz. 2D NMR spectra were zero filled in both dimensions and apodised using optimal functions chosen automatically in MNova. All urine spectra were referenced to the signals of internal TSP at 0.0 ppm and plasma spectra were referenced to the anomeric proton signal of alpha-D-glucose at 5.233 ppm. The key 950 MHz 2D ^1^H TOCSY experiments were conducted with 256 × t1 acquisitions into 2,048 data points in t2, with a relaxation delay of 2.0 s and a 90° pulse width of 8.4 us, in the pulse sequence dipsi2gpphpr. The data was zero filled to 4,096 × 1,024 data points and apodised with 90° sine square functions in both dimensions.

Interpretation of the NMR data and metabolite assignment and identification was performed using standard methods (Dona et al., 2016).

### Statistical analysis of NMR spectroscopy data

One-dimensional proton NMR data were all apodised, zero-filled, referenced (see above), baseline-corrected and phased carefully. Sections of the spectra without signals were removed in MNova; viz. 0.5 ppm to the low frequency end of the spectrum; 9.5 ppm to the high frequency end of the spectrum and the residual water signal region around 4.8 ppm. The spectra were normalised to total area of 100 and then superimposed by stacking. The spectra were then saved as a transposed, comma separated variable (csv) file and imported into Matlab 2018b (Mathworks, UK) as a cellular array.

The spectral data were aligned using the interval correlation shifting algorithm icoshift (Tomasi et al., 2011), metadata was imported and the spectra were bucketed into segments of 0.02 or 0.04 ppm width. Statistical correlation spectroscopy (STOCSY, Cloarec et al., 2005) was implemented in Matlab using a function written by Dr Kirill Veselkov (https://csmsoftware.github.io/docs/impacts/csm_stocsy.html; accessed 14.05.24).

Further statistical analysis was performed in Excel for Mac v16.88 (Microsoft USA) and in PLS-Toolbox 9.3 (Eigenvector USA) using standard methods such as principal components analysis (PCA), partial least squares discriminant analysis (PLS-DA) and support vector machines (SVM).

## Results

### FMO5 associations and phenotypes in the subjects

A linear regression additive model was used against biochemical, anthropometric and DEXA (dual-energy X-ray absorptiometry) derived fat mass measures and evaluated using the PLINK (v1.9) software (Purcell et al. 2007 PMID: 17,701,901), after adjustment for age and BMI. Two rare, exonic, non-synonymous variants were identified as polymorphic within the FMO5 gene and associated measures held within the Oxford Biobank cohort: rs58351438 and rs143837136 (Supplementary Table 1). Significant associations were seen in females for insulin with both SNPs, and additionally for SNP rs58351438, with upper body fat, especially DEXA-derived visceral fat mass (Table 1).

Both variants were then selected for recruit-by-genotype recall of Oxford Biobank participants.  10 Participants were successfully recruited heterozygous for rs58351438, however, due to its low frequency only 2 individuals heterozygous for rs143837136 were successfully recruited. 12 Wildtype matched controls were also recruited. There was no significant difference between the two groups for age and BMI or anthropometric measures (Supplementary Table 2). In line with the full Oxford Biobank cohort, there was a significant difference for Insulin in the same direction as the larger full Oxford Biobank cohort (mean ±SE, case 7.9±0.8 v control 10.9±1, p value = 0.033, Supplementary Table 2).

### Urine metabolic profiles

#### Urinary 2,3-butanediol levels (23BD)

Initial acquisition of ^1^H NMR spectroscopic data at 700 MHz indicated that there were no clear signals from 23BD in the urine or plasma samples, in contrast to the situation in *Fmo5*^*−/−*^ mice, where they are clearly visible (Veeravalli et al., 2022).

**Fig. 1:**
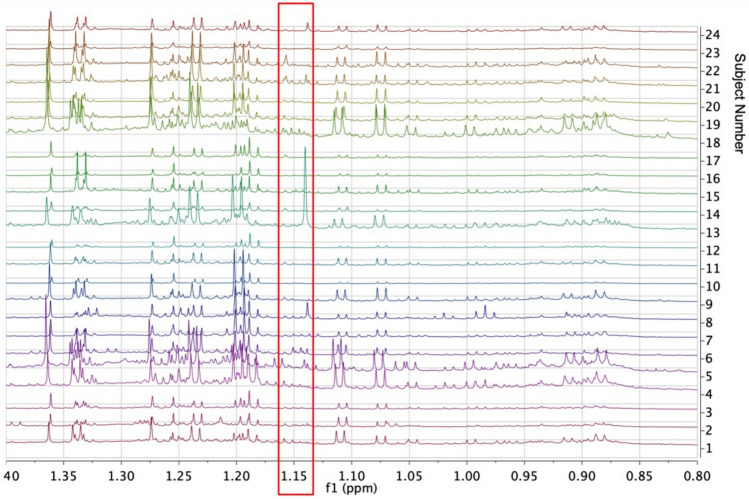
A stack plot of the 950 MHz 1D ^1^H NMR spectra of the urines of all 24 subjects in the region ca 0.8 to 1.4 ppm. The red box shows the expected positions for signals from the methyl groups of 2R, 3R-2,3-butanediol (23BD) and meso-23BD, none of which are clearly or confidently identifiable

The entire 1D ^1^H NMR data acquisition was therefore repeated at 950 MHz for the urine samples (where 23BD is more easily observed due to lack of binding to macromolecules, unlike in plasma), in order to achieve greater sensitivity of detection of the 23BD (Fig. 1). Small signals in the region of 1.15 ppm, where the complex methyl signals of 23BD are to be expected, were observed: see the spectrum for Subject 6 for instance, but these were not able to be confidently assigned to 23BD based just on 1D ^1^H NMR spectral data.

**Fig. 2:**
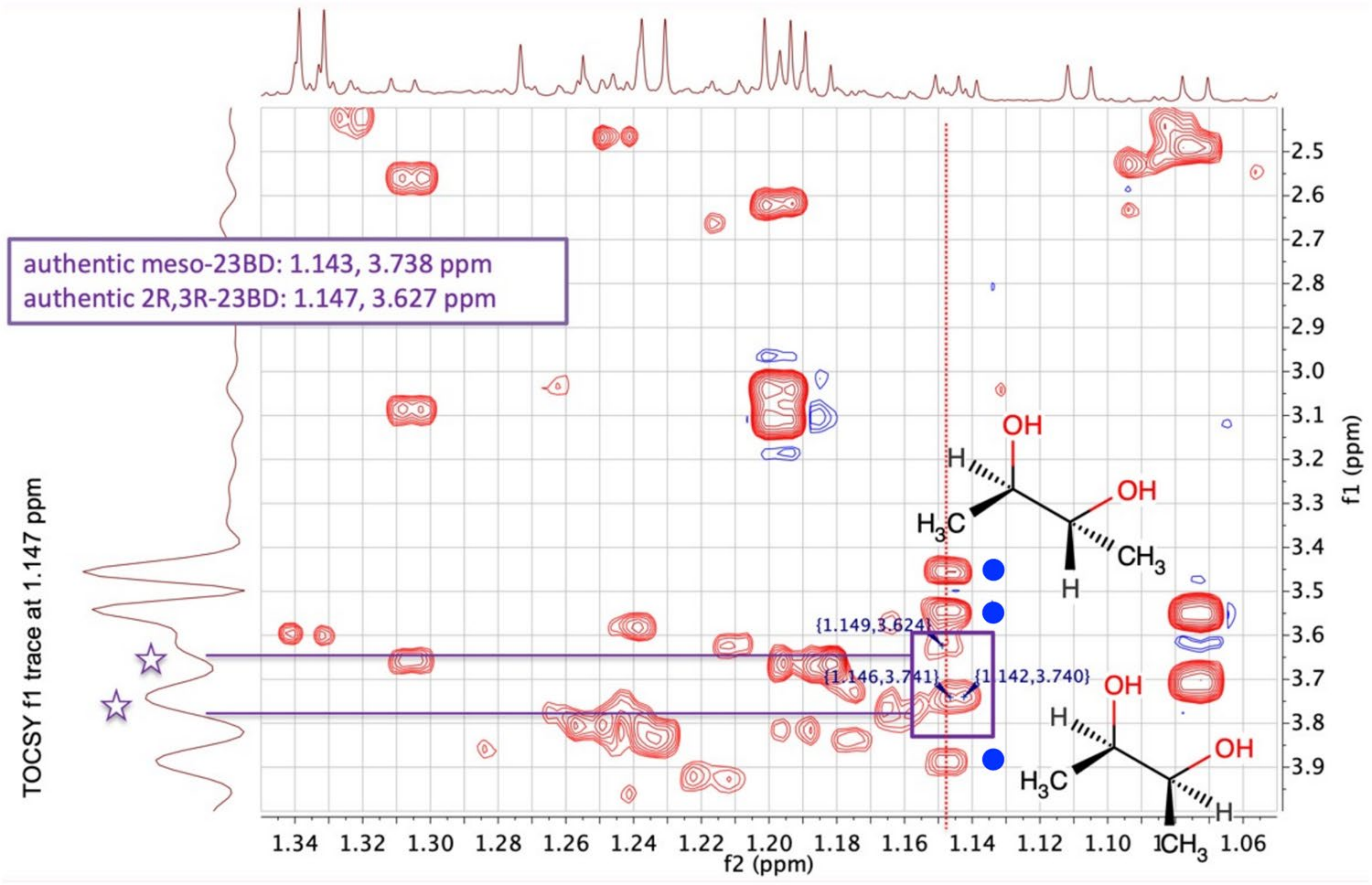
A contour plot of an expansion of the 950 MHz 2D ^1^H TOCSY NMR spectrum of the urine from Subject 6 (wildtype *FMO5*) in the f2 region ca 1.05 to 1.35 ppm. The corresponding 1D ^1^H NMR spectrum is shown on top. The contour lines in the TOCSY spectrum are cross-peaks due to connectivities between coupled hydrogens in the metabolites, such as between the methyl and methyne hydrogens in 2,3-butanediol. On the left-hand side is the f1 trace at 1.147 ppm, corresponding to the dotted red line in the 2D spectrum. The purple box on this line indicates the expected positions of the TOCSY cross-peaks for the meso- and enantiomeric isomers of 23BD, the values for which are given in the second purple box at top left. Cross-peaks for 2R, 3R-23BD and meso-23BD are observed at ca 1.149, 3.624 and 1.144, 3.741 ppm respectively, in good agreement with the expected figures for the authentic metabolites. The purple stars above the f1 trace at 1.147 indicate the cross peaks from meso- and enantiomeric 2,3-butanediol. Additional TOCSY cross-peaks are also observed from other, unknown metabolite(s) at 1.147, 3.456; 1.147, 3.543 and 1.147, 3.887 ppm; these are indicated on the figure with blue circles

2D ^1^H Total Correlation Spectroscopy (TOCSY) was therefore used to determine if the small signals at ca 1.15 ppm were connected to methyne signals at ca 3.63 and 3.73 ppm, as required for the signals to be assigned to the enantiomeric and meso-isomers of 23BD respectively. Indeed, the 2D TOCSY spectra of the urine for some of the subjects did show CH_3_ to CH cross peaks in exactly the positions expected for 23BD; for instance, Subject 6 (Fig. 2, Supplementary Fig. 1).

These small signals were confirmed as arising from 23BD on the basis of 2D heteronuclear single quantum coherence (HSQC, Supplementary Fig. 2) and 2D heteronuclear multiple bond correlation (HMBC, Supplementary Fig. 3) experiments.

**Fig. 3:**
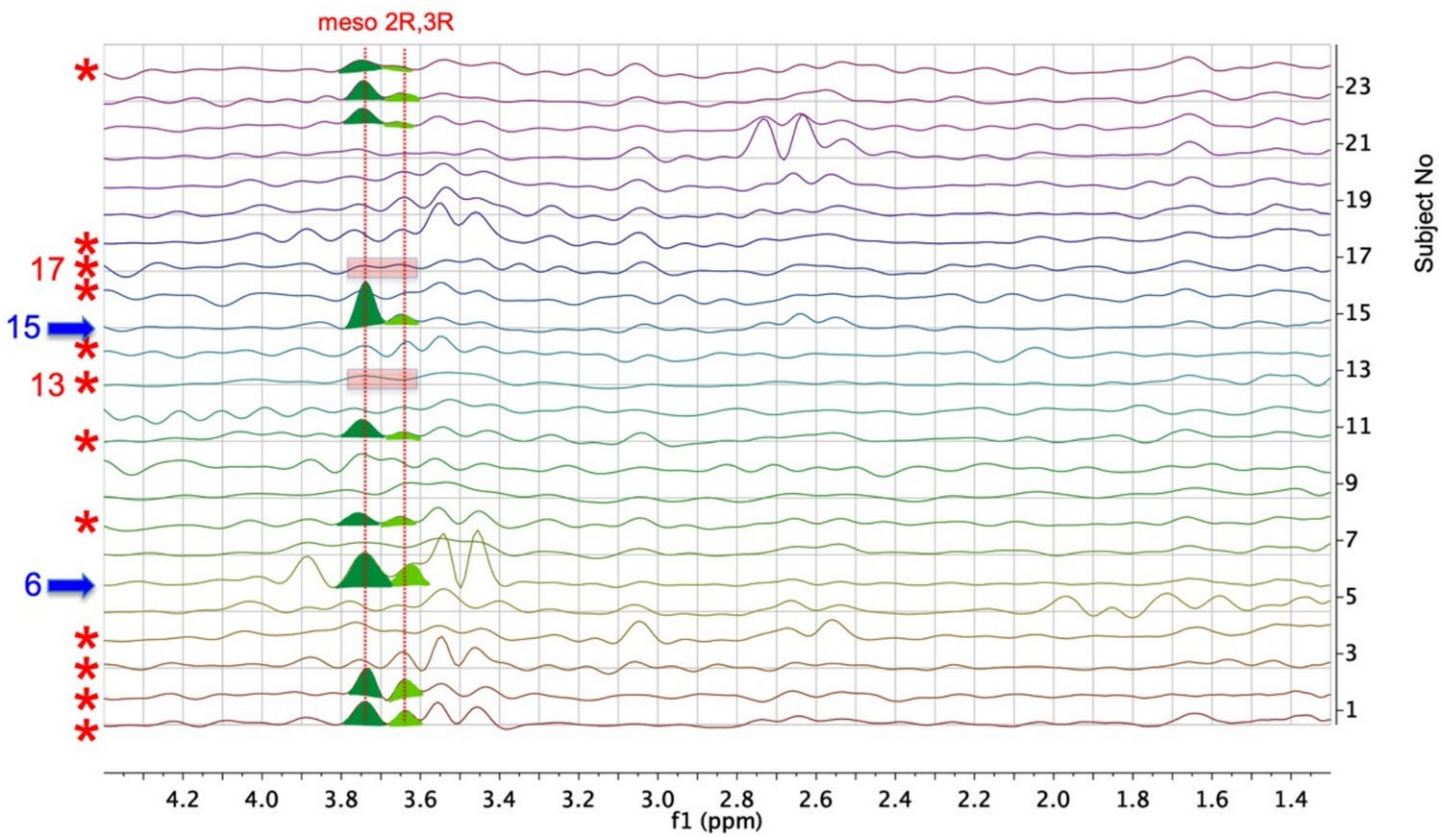
A stack plot of the f1 traces at 1.147 ppm from the 950 MHz 2D ^1^H TOCSY NMR spectra of the urines of all 24 subjects in the region ca 1.3 to 4.4 ppm. The spectra for Subjects 1 to 24 are in ascending order in the figure. Spectra from subjects with heterozygous *FMO5* genetic variants (subjects 1, 2, 3, 4, 8, 11, 13, 14, 16, 17, 18 and 24) are indicated by red asterisks. Subjects 2 and 24 had the rare rs1438371436 SNP. The peaks in these TOCSY traces arise from metabolite hydrogens with coupling connectivities to hydrogens resonating at 1.147 ppm. Vertical red lines at ca 3.63 and 3.73 ppm show the expected positions for the TOCSY cross-peaks from 2R, 3R-2,3-butanediol (23BD) and meso-23BD respectively and significant signals for these two isomers are coloured in light green and dark green respectively. Mismatches between genotype and hypothesised metabotype are clear for Subjects 6 and 15 (Subjects with wildtype *FMO5* but significant 23BD (blue arrows)), as well as for Subjects 13 and 17 (variant *FMO5* but no clear 23BD signals (pink boxes))

The f1 traces at ca 1.147 ppm of the TOCSY spectra of each of the 24 urine samples were analysed carefully for the presence or absence of signals from 23BD at ca 3.63 (enantiomeric, probably 2R, 3R-23BD) and at ca 3.74 ppm (meso-23BD) to determine the presence or absence of significant quantities of 23BD (Fig. 3).

The presence or absence of the signals of 23BD in the TOCSY f1 traces of Fig. 3 was decided based on the 5 criteria below:

1. Cross peak present for meso-23BD at ca 1.147, 3.74 ± 0.03 ppm
2. Cross peak present for 2R, 3R-23BD at ca 1.147, 3.63 ± 0.03 ppm
3. Cross peaks above the noise envelope
4. Cross-peaks well-resolved
5. If cross-peaks are shifted due to environmental conditions, pH, ionic strength etc., they should shift together, due to the chemical similarity of the 2 isomers. For example, in the f1 trace of urine sample 10 (Fig. 3) the putative 23BD cross peaks are too close in shift to be assigned as such and are likely to be due to the signals of other metabolites.

Based on these criteria, analysis of Fig. 3 gave the following results. Firstly, there is strong evidence for the presence of two isomers of 23BD in the f1 traces of the urines of 9 subjects (1, 2, 6, 8, 11, 15, 22, 23, 24: Yes in Table 2) with 23BD possibly being present in 3 others (3, 14, 19: Yes? in Table 2). Secondly, 23BD appears to be absent from the urines of 8 other subjects (7, 9, 12, 13, 16, 17, 20, 21: No in Table 2) with uncertainty about 4 others (subjects 4, 5, 10 and 18: No? in Table 2).

If our hypothesis about the effect of human, heterozygous *FMO5* variation on metabolic phenotype was correct, then the 12 subjects with *FMO5* variation would be the ones to have the highest levels of 23BD in their urine, by analogy to the results found in the *Fmo5*^*−/−*^ mice. We therefore added the 9 subjects with strong evidence of the presence of 23BD to the 3 subjects with possible presence and made these 12 the candidate *FMO5* cohort*.* All other subjects were *assigned* as possessing WT *FMO5.* Somewhat surprisingly, on unblinding the study, we found that the two subjects with the largest amounts of 23BD in their urines: Subjects 6 and 15 (Fig. 3), both have a wildtype *FMO5* genotype (Fig. 3). In addition, 5 subjects in each genotype were miscalled on the basis of the NMR-detected 23BD levels (Table 2).

With the *FMO5* genotype of only 7 out of 12 subjects in each class (WT or *FMO5* variant) called correctly, our *FMO5* genotype prediction test did not reach statistical significance by Fisher’s exact test (p = 0.23). In addition, the Mann–Whitney U-test found no significant relationship between *FMO5* genotype and 23BD levels using the simple ranking of Table 2 values as follows: Yes = 3, Yes? = 2, No? = 1 and No = 0. Interestingly, but obviously not statistically significantly, the two subjects with the rarer and more-likely deleterious rs1438371436 SNP both had significant levels of 23BD in their urine.

**Table 2:**
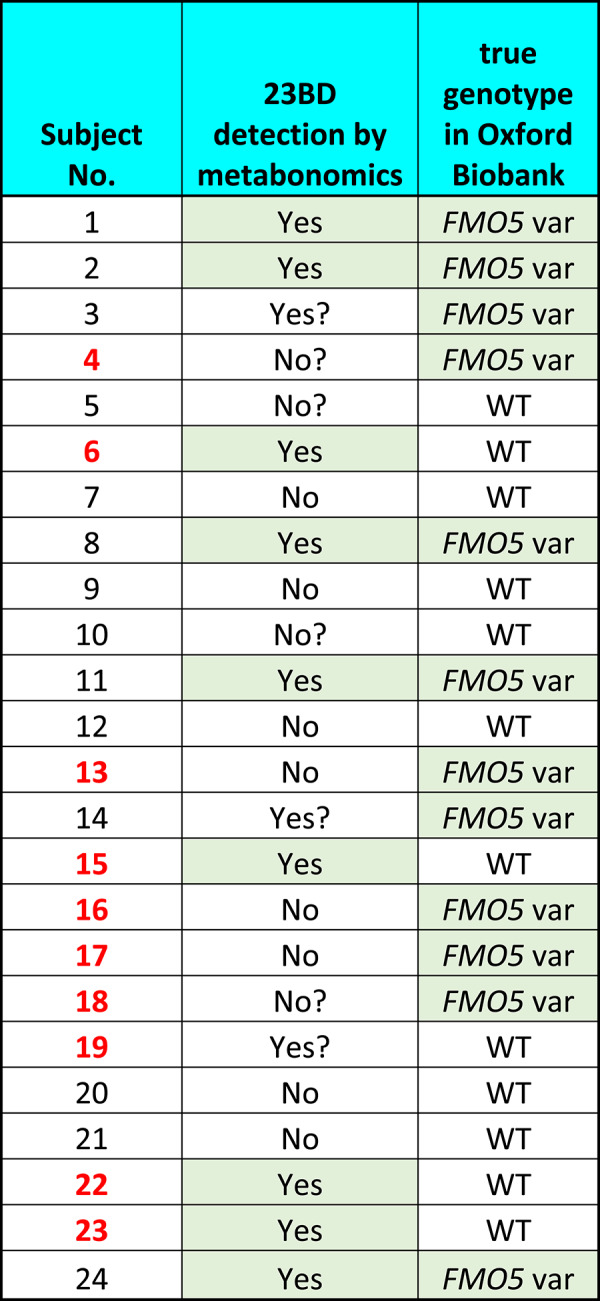
A comparison of *FMO5* genotype deduced by metabonomics detection of 23BD compared with true genotype for all 24 subjects

On the basis of the hypothesis that subjects with heterozygous *FMO5* variation should have increased 23BD, the cells are coloured green for both the gene variation (column 3) and the definitive presence of 23BD (column 2). It is clear that there are mismatches and subject numbers highlighted in bold red were incorrectly called. See text above for Column 2 definitions

#### Urinary taurine levels

An additional metabolite of interest in this study was taurine, the most abundant amino acid in the human body, albeit a sulphonic acid and remarkably, an amino acid whose final step of biosynthesis from hypotaurine is catalysed by another FMO: FMO1 (Veeravalli et al., 2020). The urinary levels of taurine in FMO5 KO mice were found to be inversely correlated to the levels of 23BD. By contrast in this study, no such relationship was observed. Relatively high taurine levels of ca 1.0 to ca 3.0 mM were detected by 950 MHz ^1^H NMR spectroscopy in the urines of Subjects 1, 4, 7, 10, 12, 15, 16, 17, 18, 19, 20 and 22 and these identifications were confirmed by the presence of the methylene to methylene cross-peaks in the corresponding TOCSY spectra. No signals for taurine were detectable in the urine spectra of subjects 2, 5, 6, 13 and 24 (Supplementary Fig. 4). Medium and low levels were detected in the urine of the other subjects. No inverse correlation was seen between the presence of high levels of 23BD in subjects 1, 2, 6, 8, 11, 15, 22, 23 and 24 and low levels of taurine. (Spearman’s Test, r = −0.022). No relationship between *FMO5* genotype and taurine level rank was found by the Mann–Whitney U test (two-tailed, p > 0.05, Supplementary Table 3). Again, tantalisingly, but not statistically significantly, the two subjects with the rarer rs1438371436 SNP, had no detectable urinary taurine (Supplementary Fig. 4).

#### Other urinary metabolites

A significant degree of variation was found in the urinary metabolic profiles of the 24 subjects, including in the levels of 4-cresol sulphate, trigonelline, mannitol and other metabolites (see Supplementary Figs. 4, 5 and 6).

#### Multivariate analyses of the urine NMR spectra

Unsupervised principal components analysis (PCA) of the bucketed (0.04 ppm intervals) NMR spectra of the urine samples at 700 MHz or 950 MHz gave no clear separation between the subjects with WT and those with heterozygous variant *FMO5* (Supplementary Fig. 7) but revealed subjects 8 and 18 as marginal outliers. Subject 8 had elevated levels of trimethylamine-*N*-oxide and subject 18 had low levels of hippurate. Removal of these WT *FMO5* outliers did not improve the group separation, nor did refining the bucket width to 0.02 ppm (data not shown).

**Fig. 4:**
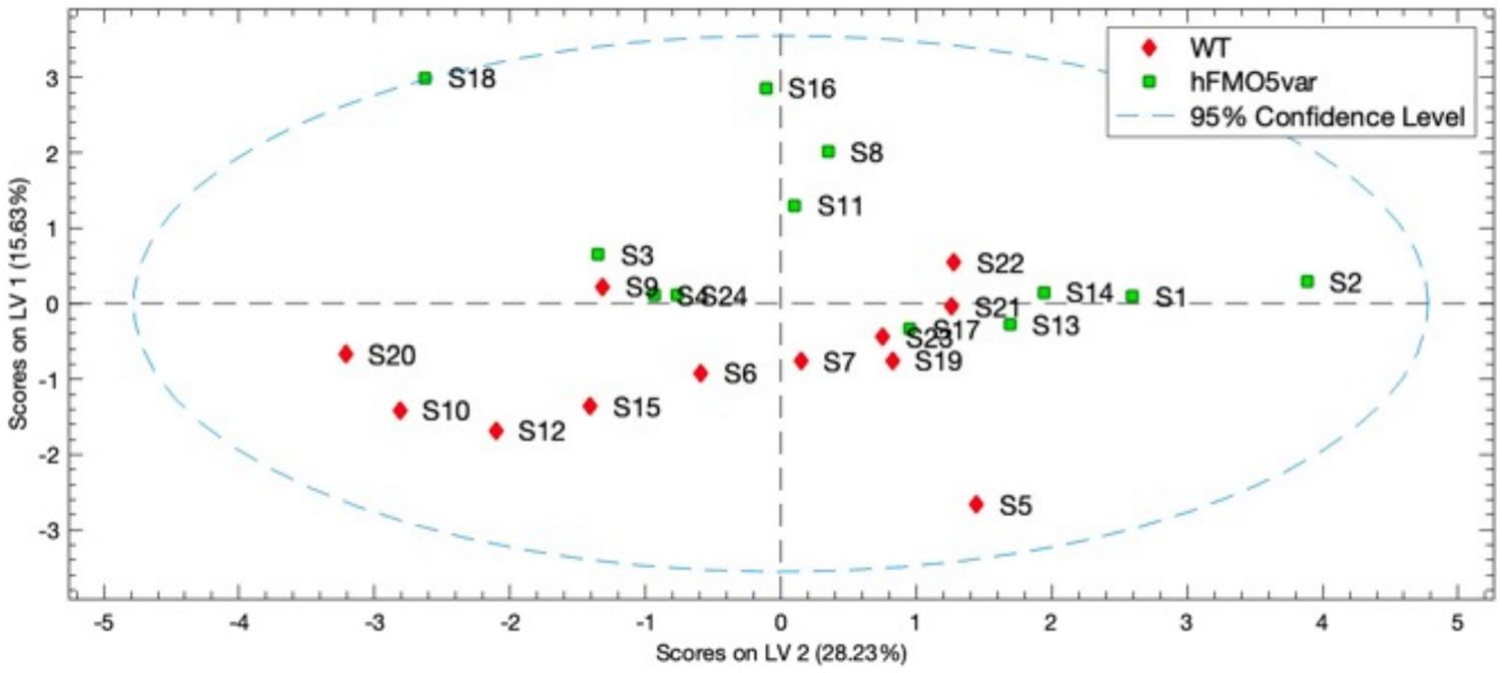
A scores plot of the mean centre scaled, partial least squares – discriminant analysis (PLS-DA) of the 700 MHz 1D ^1^H NMR spectra of the urines of all 24 subjects, with buckets set at 0.02 ppm width, showing latent variable 1 (LV1) plotted against LV2. Subjects 2 and 24 had the rare rs1438371436 SNP. A partial group separation across LV1 was achieved in the model, with most wildtype (WT) samples having negative LV1 values

Supervised multivariate analysis with partial least squares– discriminant analysis (PLS-DA), using buckets of 0.02 ppm width, showed a partial separation of the groups for 700 MHz (Fig. 4) and 950 MHz NMR urine data (Supplementary Fig. 8).

For the WT subjects, the true positive rate (TPR) and the false positive rate (FPR) in the model were 83% (10/12) and 8% (1/12) respectively and for the *FMO5* variant subjects the TPR and FPR were 92% and 17% respectively for the 700 MHz data; similar results were obtained at 950 MHz (Supplementary Fig. 8). Cross validation using Venetian blinds (10 Data Splits, Max no of LVs 20, 2 samples per blind (thickness = 1 and average left out data 10% (8 to 13% range))) gave TPR and FPR for WT as 58% and 33% respectively and TPR and FPR for *FMO5* variant subjects as 67 and 42% respectively. Orthogonal-PLS-DA gave a slightly improved looking separation, but the model statistics were identical. Cross validation was performed with Venetian Blinds using 10 data splits, 1 sample per blind and an average of 10% of data left out at each with resulting R^2^ of 0.60 and Q^2^ of 0.06, the latter indicating a lack of predictivity in the model.. Analysis of the loadings for the model showed that the most significant positive features in LV1 were due to creatinine (3.044 and 4.054 ppm), and TMAO (3.276 ppm), whilst the most significant negative features were due to an unknown (7.110 ppm) and citrate (2.656 and 2.519 ppm).

#### Statistical correlation spectroscopy (STOCSY) analysis of the 950 MHz ^1^H NMR spectra of the urine samples

STOCSY analysis showed several interesting metabolic correlations in the ^1^H NMR spectra of the urine samples, the most significant of which are summarised in Supplementary Table 4.

### Plasma metabolite analysis

The ^1^H NMR spectra of human plasma have sharp, well resolved signals from freely moving small metabolites superimposed on very broad signals from large, slow-moving macromolecules such as lipoproteins (Wurtz et al., 2017). The 700 MHz 1D 1H NMR spectra of the plasma of the subjects had less variance than those of the urine, as would be expected, due to homeostatic control (Supplementary Fig. 9). No clear signals from either 2,3-butanediol or taurine were observed in any of the plasma spectra. There are some variations in the levels of ethanol across the spectra and subject 5 had much higher lipoprotein signals than all the other subjects. No significant group separation was achieved by unsupervised (PCA) or supervised methods (PLS-DA or support vector machines; data not shown).

## Discussion

### Overview

*Fmo5*^*−/−*^ Mice have a lean phenotype (low cholesterol and body fat) and slow metabolic ageing amongst other characteristics (Veeravalli et al., 2022). The metabolic phenotype of *Fmo5*^*−/−*^ mice is characterised by high levels of two isomers of 2,3-butanediol; an enantiomeric isomer (thought to be the 2R, 3R-isomer) and the meso isomer. The meso isomer is the major isomer and the meso- to enantiomeric- isomer ratio increases with age from 15 to 60 weeks (Veeravalli et al., 2022), amongst other changes (Varshavi et al., 2018). No 23BD is detected normally in WT mice. Treatment of WT mice with 23BD gave rise to a low cholesterol, low epididymal fat phenotype, similar to that seen in the *Fmo5*^*−/−*^ mice. The production of 23BD via the altered gut microbiome of the *Fmo5*^*−/−*^ mice, is consistent with the role of FMO5 as a sensor of gut bacteria (Scott et al., 2017). However, fecal transplant from *Fmo5*^*−/−*^ mice to WT mice did not induce the low cholesterol, low epididymal fat phenotype and it is now thought that the production of 23BD occurs in the stomach (Veeravalli et al., 2022). Given the need to find an effective and safe anti-obesogenic agent for a world population where obesity incidence is increasing rapidly (Lin & Li, 2021) we wished to understand if genetic variation in human *FMO5* would lead to any of the clinical and metabolic phenotype changes seen in the *Fmo5*^*−/−*^ mice.

In order to test the 3 aims of this study (see Introduction), 12 female volunteers with variations in their FMO5, together with 12 gender- and age-matched controls were recruited from the Oxford Biobank (Karpe et al., 2018). These 24 subjects were studied to determine if there were statistically significant differences in: (1) their clinical profiles; (2) their excretion of 23BD and (3) their broader metabolite profiles.

### Clinical profiles of subjects with coding variations in FMO5

Statistical analysis of individuals heterozygous for the rs58351438 and rs143837136 FMO5 polymorphisms within the Oxford Biobank cohort showed significant associations with insulin levels. Additionally, rs58351438 showed significant associations with anthropometric and DEXA-derived upper body fat measures (Table 1). Analysis of the 12 case subjects with heterozygous coding variations in *FMO5* combined together*,* compared to their matched controls, although a small dataset, also showed a statistically significant lowering of insulin levels, p = 0.033, and a trend to lower visceral fat, p = 0.11 (Supplementary Table 2). This gave further encouraging support to the notion of translation of the *Fmo5*^*−/−*^ mouse phenotype to humans.

### 23BD metabolite profiles in subjects with coding variations in FMO5

In contrast to the situation in mice, where there is a clear distinction between high levels of urinary 23BD observed simply by one-dimensional (1D) ^1^H NMR spectroscopy in male *Fmo5*^*−/−*^ mice and an absence in the WT mice (Veeravalli et al., 2022), no clear observations of 23BD could be seen in the 1D ^1^H NMR spectra of the urines of the female subjects of this study, even at a field strength of 22.3 Tesla (Fig. 1). This is almost certainly not due to a gender issue, as high levels of 23BD are equally clearly observed in the urine of female *Fmo5*^*−/−*^ mice (Everett, Shephard et al., unpublished data).

Using more sophisticated 2D NMR methods, 23BD could be unequivocally identified in the urines of a number of the female subjects (Figs. 2, 3). However, there was no clearcut correlation between the subject’s *FMO5* genotype and the presence of high, low or no levels of 23BD in the urine (Table 2). Using high levels of urinary 23BD to ‘call’ the *FMO5* variant genotype, five wrong calls were made for each of two genotypes; *FMO5* variant and WT. As a consequence, our *FMO5* genotype prediction test based on 23BD levels fails Fisher’s exact method for statistical significance of calling the classes correctly, with calculated p = 0.23. This failure to correlate human urinary 23BD levels with heterozygous *FMO5* genotype is clearly exemplified by Subjects 6 and 15. These two subjects had the highest 23BD levels of any of the subjects, but both had the WT *FMO5* genotype*.*

The failure to correlate urinary 23BD levels with *FMO5* genotype can be explained on the basis of the following factors: (i) the subjects were heterozygous for the variation in *FMO5* and each one of them still had one fully working copy of the enzyme, in contrast to the situation in *Fmo5*^*−/−*^ mice where both copies were ablated; (ii) the human subjects on this study likely had more overall genetic variation between them than the inbred C57BL/6 J mice used in the *Fmo5*^*−/−*^ study; (iii) the human subjects were not kept in controlled laboratory conditions, (iv) the human subjects did not have a standardised diet, in contrast to the standard chow fed to the mice and (v) we had quite limited numbers of subjects due to the rarity of the SNPs. We hypothesise that the consequence of factor (i), the heterozygous nature of the variation in human *FMO5,* would be to reduce the penetrance of the coding variations. We also hypothesise that the impact of factors (ii), (iii) and (iv) would be to create greater intra-group genetic and microbiome diversity, such that variations in microbiome-produced 23BD levels are not clearly related to *FMO5* genotype.

### Broader metabolite profiles of subjects with coding variations in FMO5

In contrast to the finding of an inverse correlation between the levels of urinary 23BD and taurine in *Fmo5*^*−/−*^ mice, no such correlation was found for the urines of subjects with either WT or variant *FMO5*. Two of the subjects with the highest levels of 23BD, subjects 1 and 15, also had high levels of taurine, whilst two of the subjects, 5 and 13, with no clear signals for 23BD also had no clearly detectable taurine. There was also no correlation between *FMO5* genotype and taurine levels.

The large intra-group variance in metabolite profiles prevented any clear separation of the WT and heterozygous *FMO5* genotypes using unsupervised multivariate methods such as principal components analysis (PCA, Supplementary Fig. 7). A partial group separation between subjects with WT or heterozygous *FMO5* was achieved using PLS-DA (Fig. 4) but the cross-validation was not strong.

## Conclusions

Statistical analysis of individuals heterozygous for the rs58351438 and rs143837136 FMO5 polymorphisms within the Oxford Biobank female cohort showed significant association with anthropometric and Dexa-derived upper body fat measures along with insulin levels. However, heterozygous coding variation of human *FMO5* did not replicate the 23BD metabotype seen in the *Fmo5*^*−/−*^ mice. It is possible that human *FMO5* heterozygosity represents a recessive model, although we recognise that our low metabonomics subject numbers limit definitive conclusions*. *Given the need for effective and safe anti-obesogenic agents and given the promising results seen on oral dosing WT mice with 23BD, further developments with this agent could be of interest.

## Supplementary Information

Below is the link to the electronic supplementary material.

Supplementary file1 (PDF 4459 KB)

## Data Availability

The original NMR spectroscopy data from this study has been deposited at MetaboLights (Yurekten et al., 2023): https://www.ebi.ac.uk/metabolights/at deposit MTBLS11120.
