# Supplementary material for: Clinical and metabolic phenotypes of Oxford Biobank subjects with variations in human flavin-containing monooxygenase 5 (FMO5)
Source: Metabolomics. 2025 Sep 9;21(5):135. doi: 10.1007/s11306-025-02308-1 (PMC12420700; doi:10.1007/s11306-025-02308-1)
Supplement: Supplementary file 1 — Supplementary file1 (PDF 4459 KB) [file 11306_2025_2308_MOESM1_ESM.pdf]

# SUPPLEMENTARY INFORMATION

# Clinical and Metabolic Phenotypes of Oxford Biobank subjects with variations in human flavin-containing monooxygenase 5 (*FMO5*)

Jeremy R. Everett[1*], Fredrik Karpe[2*], Adrien Le Guennec[3], Matt Neville[2] and Christina Redfield[4]

[1]Medway Metabonomics Research Group, University of Greenwich, Chatham Maritime, Kent, UK

[2]Oxford Centre for Diabetes, Endocrinology and Metabolism, University of Oxford, Oxford, UK and NIHR Oxford Biomedical Research Centre, OUH Foundation Trust, Oxford OX3 7LE, UK

[3]NMR Facility, Guy's Campus, King's College London, London, UK

[4]Department of Biochemistry, University of Oxford, South Parks Road, Oxford OX1 3QU, UK

* Correspondence:
Jeremy R Everett: j.r.everett@greenwich.ac.uk, ORCID ID: 0000-0003-1550-4482; Fredrik Karpe: fredrik.karpe@ocdem.ox.ac.uk, ORCID ID: 0000-0002-2751-1770

Supplementary Figure 1:

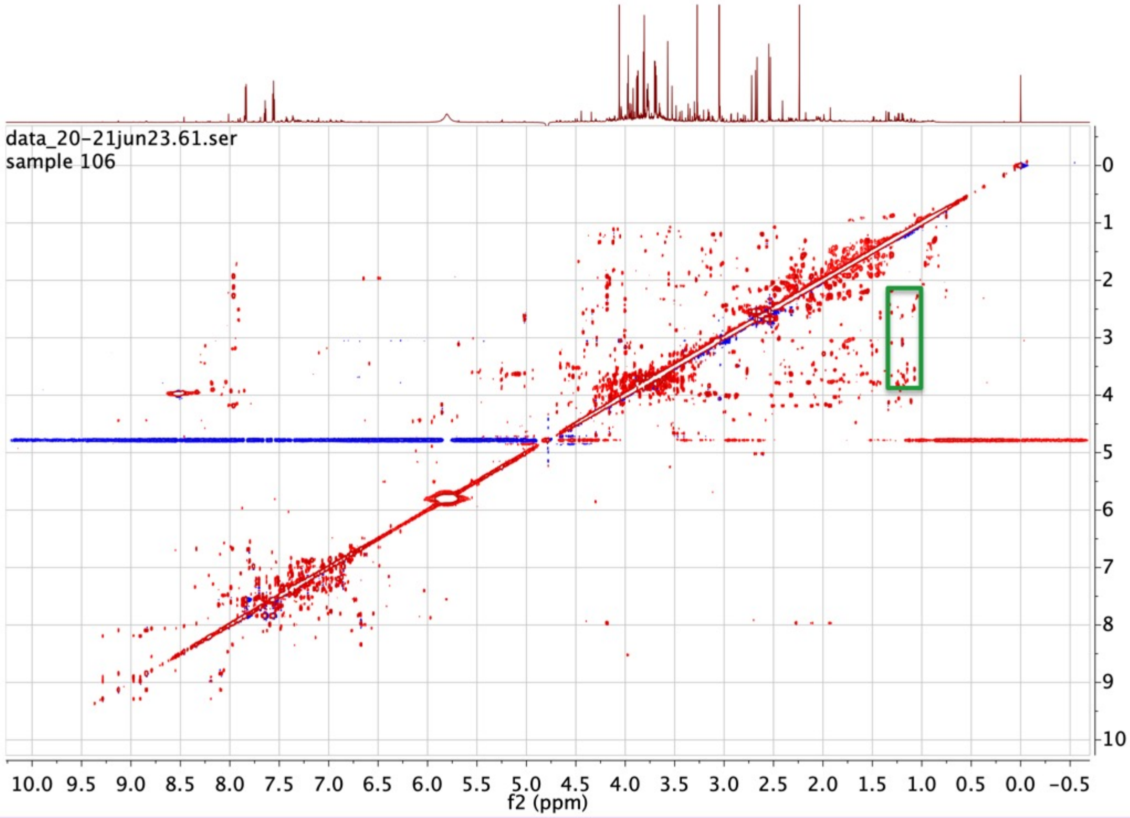


SF1. A contour plot of the 950 MHz 2D $^{1}$H TOCSY NMR spectrum of the urine of Subject 6. The corresponding 1D $^{1}$H NMR spectrum is on top. The green box highlights the region where the cross peaks of the two 23BD isomers are expected (see main text Figure 2).

Supplementary Figure 2:

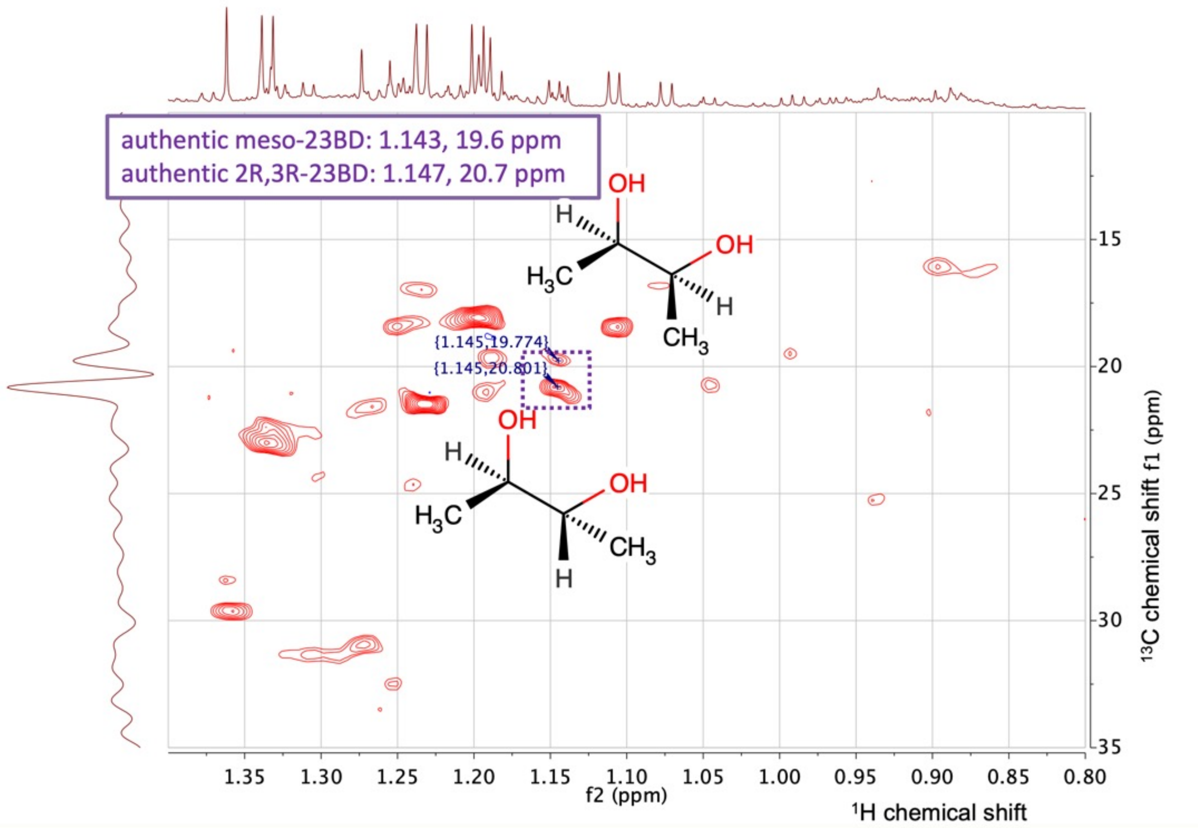


SF2. An expansion of the contour plot of the 950 MHz 2D $^1$H HSQC NMR spectrum of the urine of Subject 6 in the region expected for the signals from 23BD. The corresponding 1D $^1$H NMR spectrum is on top. The solid purple box gives the HSQC cross peak positions for authentic 23BD. In the dashed purple box, two cross peaks are observed at 1.145, 19.8 and 1.145, 20.8 ppm, in good agreement with the values expected for the two isomers of 23BD. The f1 trace at 1.145 ppm is shown on the left side.

Supplementary Figure 3:

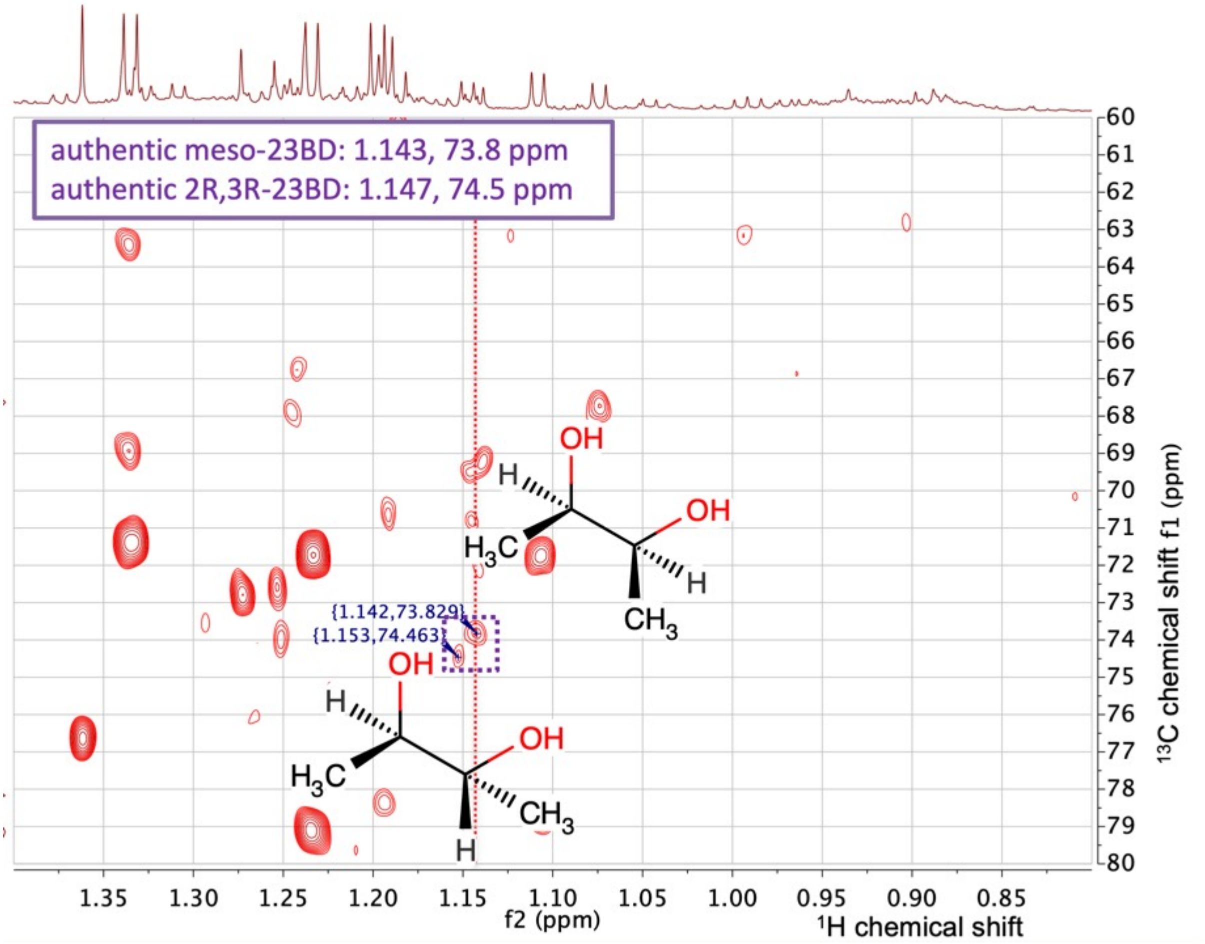


SF3. An expansion of the contour plot of the 950 MHz 2D $^{1}$H HMBC NMR spectrum of the urine of Subject 6 in the region expected for the signals from 23BD. The corresponding 1D $^{1}$H NMR spectrum is on top. The solid purple box gives the 2/3-bond HMBC cross peak positions for authentic 23BD. In the dashed purple box, two cross peaks are observed at 1.142, 73.8 and 1.153, 74.5 ppm, in good agreement with the values expected for the two isomers of 23BD.

Supplementary Figure 4:

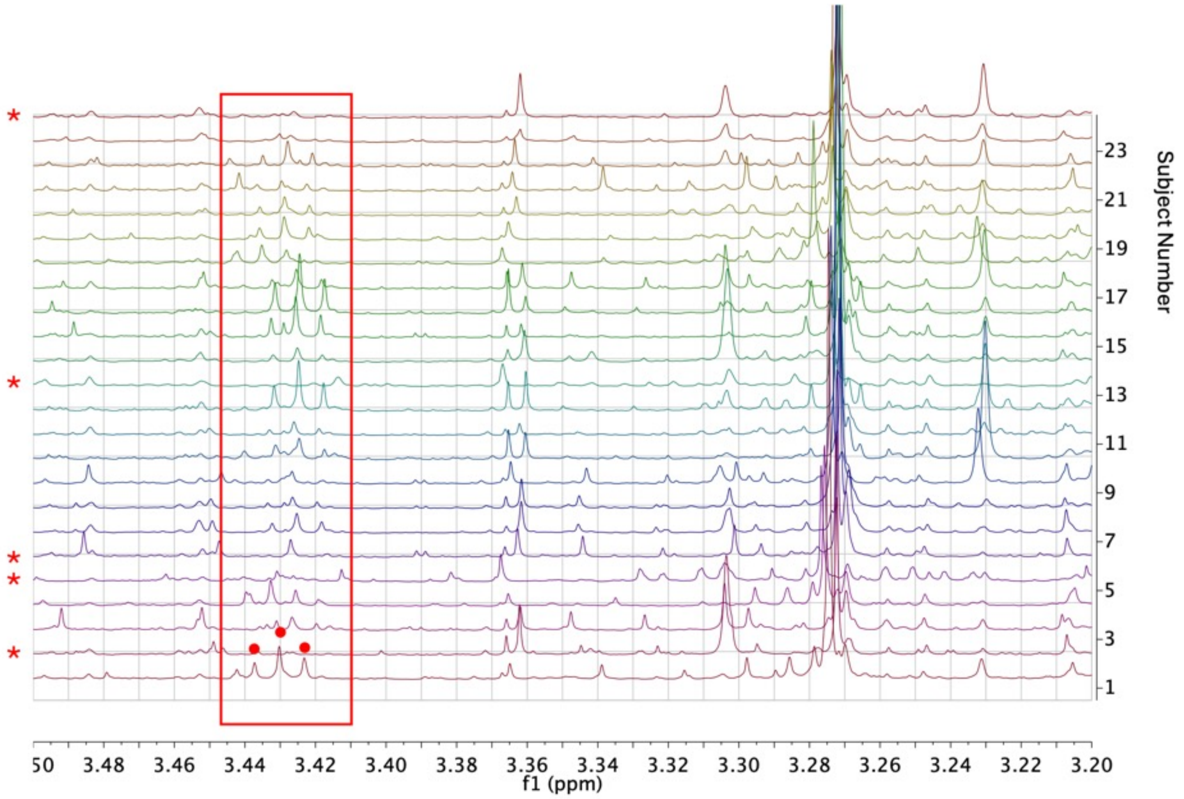


SF4. An expansion of the 950 MHz $^1$H NMR spectra of the urines of the 24 subjects in the region of the two triplet signals from taurine at ca 3.43 and ca 3.28 ppm. Red asterisks mark the spectra of those urines with no detectable signals from taurine; viz Subjects 2, 5, 6, 13 and 24. For clarity, red dots mark each of the transitions of the triplet at ca 3.43 pm in the spectrum of the urine of Subject 1. Some variability in the chemical shift of this signal across the 24 spectra is clear. The triplet signals at ca 3.28 ppm are partly obscured by overlapping resonances from trimethylamine-*N*-oxide and other metabolites.

Supplementary Figure 5:

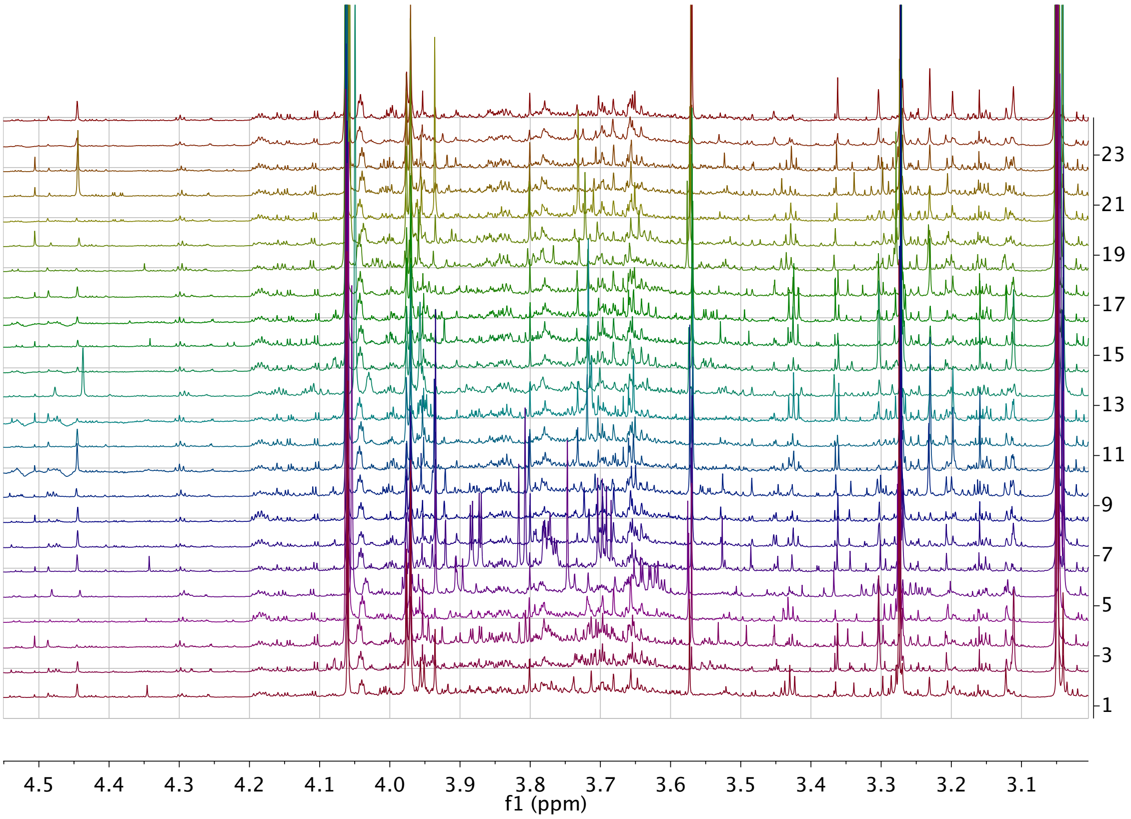


SF5. An expansion of the 950 MHz $^1$H NMR spectra of the urines of the 24 subjects in the region ca 4.55 to 3.00 ppm. Significant inter-subject variability is apparent in the levels of trigonelline (singlet at ca 4.45 ppm) and especially mannitol, with multiplets at 3.70, 3.78, 3.81 and 3.88 ppm being especially evident in the spectrum of subject 6, but also present in the spectrum of subject 3's urine, but no others.

Supplementary Figure 6:

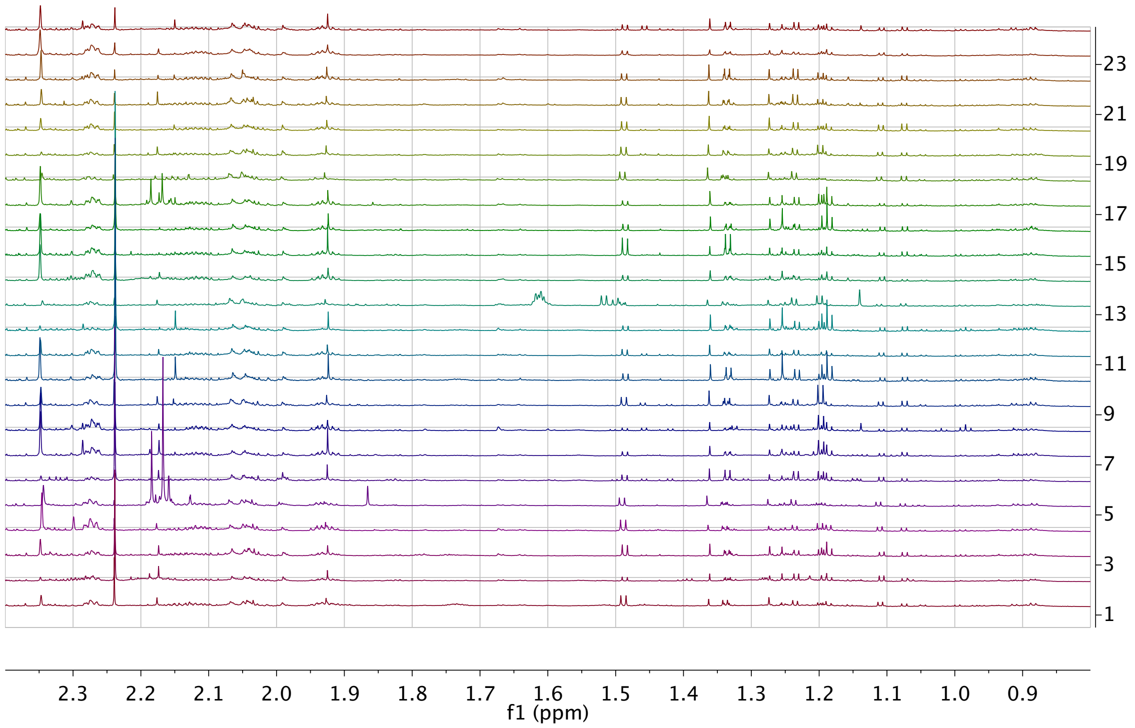


SF6. An expansion of the 950 MHz $^{1}H$ NMR spectra of the urines of the 24 subjects in the region ca 2.4 to 0.8 ppm. Significant inter-subject variability is apparent in the levels of 4-cresol sulphate (singlet at ca 2.35 ppm) and the acetyl singlets at 2.17 ppm, especially evident in the spectra of subjects 5 and 17; but also the unknown multiplet at ca 1.61 ppm present in the spectrum of subject 13's urine, but no others.

Supplementary Figure 7:

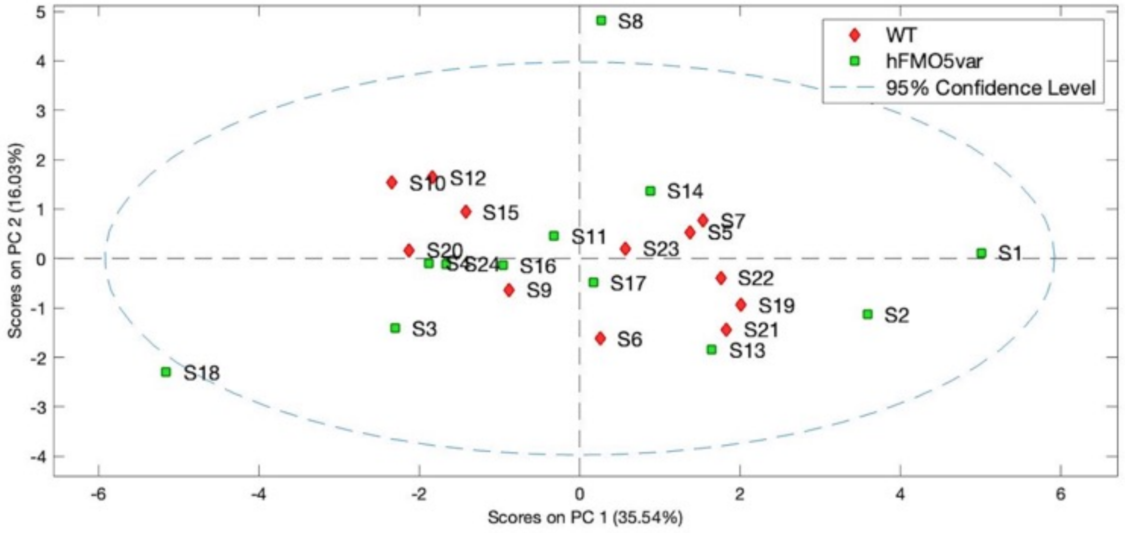


SF7. A mean centre scaled scores plot of the PCA of the 700 MHz $^1$H NMR spectra of the urines of the 24 subjects, bucketed to 0.04 ppm intervals. No clear inter-group separation is apparent between the subjects with WT human *FMO5* (red diamonds) and those subjects heterozygous for variant human *FMO5* (green squares). Subjects 8 and 18 are minor outliers.

Supplementary Figure 8:

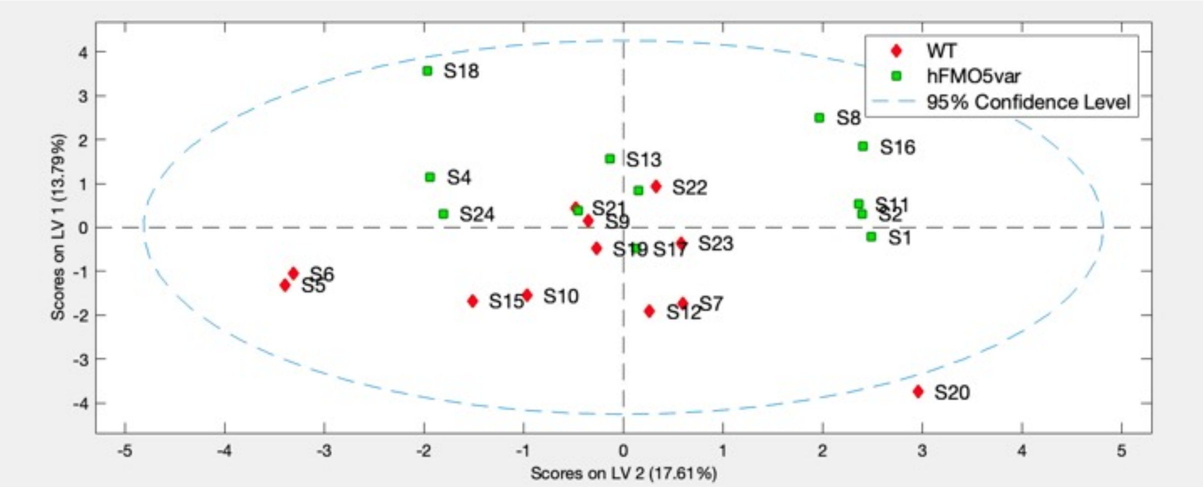


SF8. A mean centre scaled scores plot of the PLS-DA of the 950 MHz $^{1}$H NMR spectra of the urines of the 24 subjects, bucketed to 0.02 ppm intervals. A partial group separation is apparent between the subjects with WT human *FMO5* (red diamonds) and those subjects heterozygous for variant human *FMO5* (green squares). Subject 20 is a minor outlier.

Cross validation with Venetian Blinds, left out data 8 to 13%, average left out 10%, showed that the model went from true positive rates of 83% for both WT and hFMO5var to 67 and 58 % respectively, similar to the 700 MHz model. The loadings of LV1 were similar to those at 700 MHz (see main text).

Supplementary Figure 9:

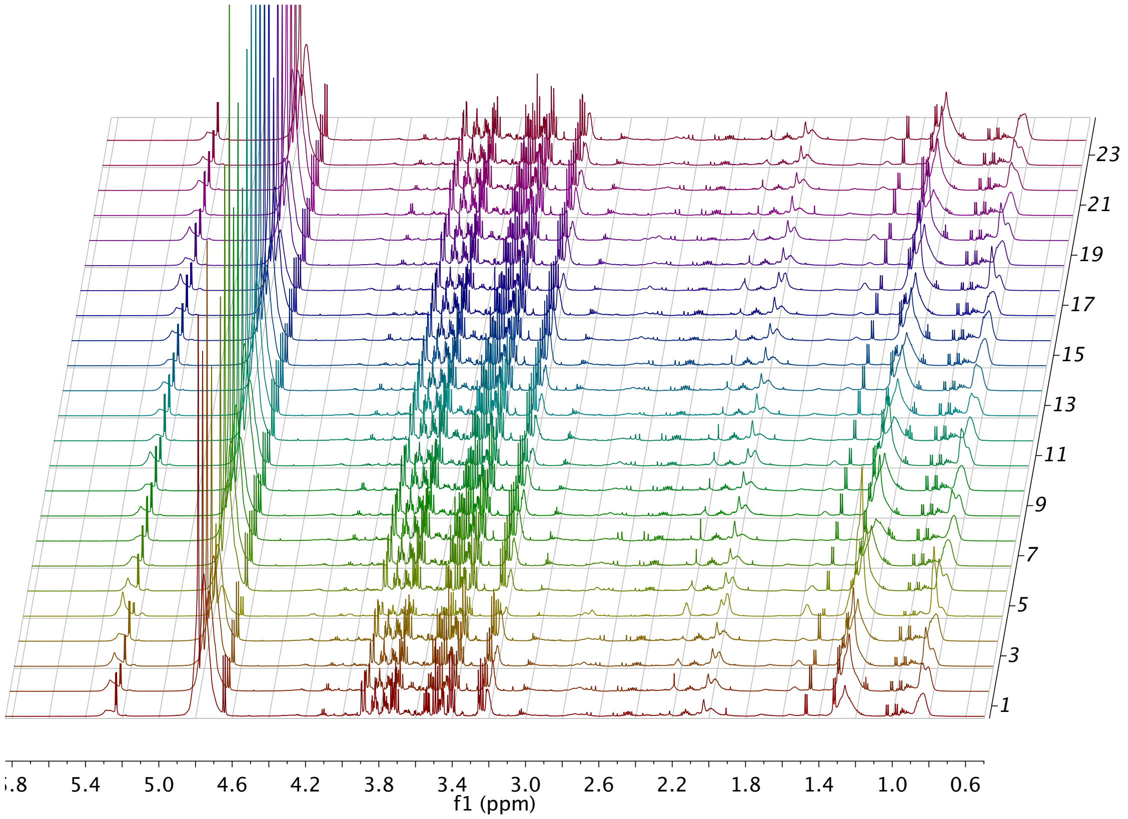


SF9. A stack plot of the low frequency region of the 700 MHz CPMG spin-echo $^{1}$H NMR spectra of the plasma of the 24 subjects numbered on the right side of the plot.

Supplementary Table 1. Characteristics of the variations of interest in the Study Subjects

| Variation ID | Position* | Variant | CDNA Position | AA Position | AA Change | MAF | Predicted impact** |
|---|---|---|---|---|---|---|---|
| rs58351438 | chr1:146684095 | T\|C | 496 | 166 | K>E | 0.008 | Neutral (75 %) |
| rs143837136 | chr1:146658604 | C\|T | 1477 | 493 | D>H | 0.001 | Deleterious (61%) |

* Coordinates are based on Genome assembly GRCh37/hg19

** Predicted impact is based on the consensus score from the PredictSNP Consensus prediction tool (Bendl *et al.* 2014,PMID: 24453961), the expected accuracy of the prediction is in brackets.

Supplementary Table 2: Analysis of the Clinical Phenotypes of the Case and Control Subjects in this Study

| | Mean ± SE | | | |
|---|---|---|---|---|
| | case (n=12) | control (n=12) | *p* value* | *p* value** |
| Age | 51.1±2.2 | 53.8±2.9 | 0.485 | 0.4683 |
| BMI | 23.9±0.7 | 22.5±0.7 | 0.2242 | 0.1782 |
| Insulin | 7.9±0.8 | 10.9±1 | 0.0659 | 0.033 |
| Glucose | 5±0.2 | 5±0.1 | 0.6397 | |
| Waist | 78.8±1.3 | 77.8±2.7 | 0.2709 | 0.745 |
| Hip | 101.8±2.1 | 96.5±1.6 | 0.061 | 0.061 |

| | Mean ± SE | | | |
|---|---|---|---|---|
| | case (n=11) | Control (n=6) | *p* value* | *p* value** |
| Total android fat | 1291.7±164.8 | 1781±286.4 | 0.3011 | 0.2114 |
| Gynoid Fat | 4176.3±320.5 | 3761±340.7 | 0.7325 | 0.4817 |
| Visceral fat | 171.5±27.2 | 423.2±141.5 | 0.8075 | 0.1114 |
| Subcutaneous android fat | 1120.2±150.5 | 1357.7±173.7 | 0.4043 | 0.4077 |

Statistical testing performed in Graphpad Prism v10.4.1
* Significance based on Mann-Whitney U test
** Significance based on unpaired 2-tailed, t-test, where data passes the test for normality (glucose data was not normally distributed)

Supplementary Table 3: Human urinary taurine levels in 950 MHz 1H NMR spectra

| Sample No | Taurine Level | Genotype | 23BD Level | Rank Taurine | Rank Genotype | Rank 23BD |
|---|---|---|---|---|---|---|
| 24 | 0 | 2 | 3 | 21.5 | 6.5 | 5 |
| 23 | 6 | 1 | 3 | 18 | 18.5 | 5 |
| 22 | 19 | 1 | 3 | 5.5 | 18.5 | 5 |
| 21 | 8 | 1 | 0 | 17 | 18.5 | 20.5 |
| 20 | 14 | 1 | 0 | 12 | 18.5 | 20.5 |
| 19 | 18 | 1 | 2 | 7 | 18.5 | 11 |
| 18 | 15 | 2 | 1 | 10 | 6.5 | 14.5 |
| 17 | 15 | 2 | 0 | 10 | 6.5 | 20.5 |
| 16 | 44 | 2 | 0 | 1 | 6.5 | 20.5 |
| 15 | 30 | 1 | 3 | 3 | 18.5 | 5 |
| 14 | 11 | 2 | 2 | 13 | 6.5 | 11 |
| 13 | 0 | 2 | 0 | 21.5 | 6.5 | 20.5 |
| 12 | 37 | 1 | 0 | 2 | 18.5 | 20.5 |
| 11 | 10 | 2 | 3 | 14.5 | 6.5 | 5 |
| 10 | 16 | 1 | 1 | 8 | 18.5 | 14.5 |
| 9 | 0 | 1 | 0 | 21.5 | 18.5 | 20.5 |
| 8 | 9 | 2 | 3 | 16 | 6.5 | 5 |
| 7 | 15 | 1 | 0 | 10 | 18.5 | 20.5 |
| 6 | 0 | 1 | 3 | 21.5 | 18.5 | 5 |
| 5 | 0 | 1 | 1 | 21.5 | 18.5 | 14.5 |
| 4 | 19 | 2 | 1 | 5.5 | 6.5 | 14.5 |
| 3 | 10 | 2 | 2 | 14.5 | 6.5 | 11 |
| 2 | 0 | 2 | 3 | 21.5 | 6.5 | 5 |
| 1 | 24 | 2 | 3 | 4 | 6.5 | 5 |

Taurine level measured by Mnova peak height of central part of triplet at ca 3.43 ppm in normalised 950 MHz 1H NMR spectra x1,000

Genotype 1 = WT hFMO5; 2 = hFMO5 variant

23BD level: 3 = detected; 2 = probably present; 1 = probably not present and 0 = not detected

Supplementary Table 4: Significant Positive (+) or Negative (-) Statistical Correlation Spectroscopy (STOCSY) signals from the 950 MHz $^{1}$H NMR spectra of the Urine Samples

| **target metabolite (peak)** | **STOCSY correlations** |
|---|---|
| ethanol (1.19 ppm) | formate (+), taurine (+), acetone (+) and unknown (s, 1.254 ppm: +) |
| citrate (2.54 ppm) | cis-aconitate (+); acetate (+) and hippurate (-) |
| creatinine (3.05 ppm) | unknown (t, 2.6 Hz, 4.04 ppm; +), hippurate (-) and phenylacetylglutamine (-) |
| TMAO (3.272 ppm) | unknown (1.02 ppm; +) and unknown (t, 0.98 ppm; +) |
| taurine (3.43 ppm) | formate (+); acetone (+); unknown (s, 1.26 ppm; +) and ethanol (+) |
| hippurate (7.56 ppm) | citrate (-) |
| formate (8.46 ppm) | unknown (s, 3.74 ppm; +); taurine (+); unknown (s, 3.16 ppm;+); citrate (+), unknown (s, 1.26 ppm; +) and ethanol (+) |

Footnote: s = singlet; t = triplet; TMAO = trimethylamine-*N*-oxide
